# Supplementary material for: From sequence to enzyme mechanism using multi-label machine learning
Source: BMC Bioinformatics. 2014 May 19;15:150. doi: 10.1186/1471-2105-15-150 (PMC4229970; doi:10.1186/1471-2105-15-150)
Supplement: Additional file 2 — Java code of ml2db. Additional file ml2db_code.tar.gz contains the Java source code to run the multi-label machine learning experiments and save the results to database. The code’s Javadoc is included. [file 1471-2105-15-150-S2.zip › additional file 2/ml2db/ml2db/doc/mulan/examples/GettingPredictionsOnUnlabeledData.html]

GettingPredictionsOnUnlabeledData


JavaScript is disabled on your browser.


- Overview
- Package
- Class
- Use
- Tree
- Deprecated
- Index
- Help

- Prev Class
- Next Class

- Frames
- No Frames

- All Classes

- Summary:
- Nested |
- Field |
- Constr |
- Method

- Detail:
- Field |
- Constr |
- Method


mulan.examples

## Class GettingPredictionsOnUnlabeledData

- java.lang.Object
- - mulan.examples.GettingPredictionsOnUnlabeledData

- ---

    

  ```
  public class GettingPredictionsOnUnlabeledData
  extends java.lang.Object
  ```

  This examples shows how you can retrieve the predictions of a model on
  unlabeled data. Unlabeled multi-label datasets should have the same
  structure as the training data. The actual values of the labels could be
  either unspecified (set to symbol ?), or randomly set to 0/1.

  Version:
  :   2010.12.15

  Author:
  :   Grigorios Tsoumakas

- - ### Constructor Summary

    Constructors

    Constructor and Description

    `GettingPredictionsOnUnlabeledData()`
  - ### Method Summary

    Methods

    | Modifier and Type | Method and Description |
    | --- | --- |
    | `static void` | `main(java.lang.String[] args)` Executes this example |

    - ### Methods inherited from class java.lang.Object

      `equals, getClass, hashCode, notify, notifyAll, toString, wait, wait, wait`

- - ### Constructor Detail


    - #### GettingPredictionsOnUnlabeledData

      ```
      public GettingPredictionsOnUnlabeledData()
      ```
  - ### Method Detail


    - #### main

      ```
      public static void main(java.lang.String[] args)
      ```

      Executes this example

      Parameters:
      :   `args` - command-line arguments -arff, -xml and -unlabeled


- Overview
- Package
- Class
- Use
- Tree
- Deprecated
- Index
- Help

- Prev Class
- Next Class

- Frames
- No Frames

- All Classes

- Summary:
- Nested |
- Field |
- Constr |
- Method

- Detail:
- Field |
- Constr |
- Method
